# Supplementary material for: Hot spot prediction in protein-protein interactions by an ensemble system
Source: BMC Syst Biol. 2018 Dec 31;12(Suppl 9):132. doi: 10.1186/s12918-018-0665-8 (PMC6311905; doi:10.1186/s12918-018-0665-8)
Supplement: Supplementary file 2 — All relevant MATLAB code. A simple Matlab implement of our predictor. (ZIP 19,742 kb) [file 12918_2018_665_MOESM2_ESM.zip › Additional file 2/Read ASA/ASA website/dbMPIKT/3BIWA.html]

 NetSurfP - Protein Surface Accessibility and Secondary Structure Predictions

  

|  |  |  |
| --- | --- | --- |
|  |  | NetSurfP - Protein Surface Accessibility and Secondary Structure Predictions  Technical University of Denmark |

  


---

```
# For publication of results, please cite:
# A generic method for assignment of reliability scores applied to solvent accessibility predictions.
# Bent Petersen, Thomas Nordahl Petersen, Pernille Andersen, Morten Nielsen and Claus Lundegaard
# BMC Structural Biology 2009, 9:51 doi:10.1186/1472-6807-9-51
#
# Column 1: Class assignment - B for buried or E for Exposed - Threshold: 25% exposure, but not based on RSA
# Column 2: Amino acid
# Column 3: Sequence name
# Column 4: Amino acid number
# Column 5: Relative Surface Accessibility - RSA
# Column 6: Absolute Surface Accessibility
# Column 7: Z-fit score for RSA prediction
# Column 8: Probability for Alpha-Helix
# Column 9: Probability for Beta-strand
# Column 10: Probability for Coil
E A  3BIWA                  1    0.701  77.250  -2.423   0.003   0.003   0.994
E D  3BIWA                  2    0.654  94.241  -0.840   0.018   0.047   0.935
E P  3BIWA                  3    0.356  50.545  -1.575   0.053   0.043   0.903
E Q  3BIWA                  4    0.540  96.355  -0.471   0.053   0.043   0.903
E K  3BIWA                  5    0.596 122.659  -0.300   0.053   0.043   0.903
B L  3BIWA                  6    0.240  43.907  -0.551   0.053   0.043   0.903
E D  3BIWA                  7    0.643  92.714   0.164   0.018   0.047   0.935
E D  3BIWA                  8    0.624  89.875  -0.967   0.018   0.047   0.935
E V  3BIWA                  9    0.584  89.684  -0.284   0.018   0.047   0.935
E D  3BIWA                 10    0.483  69.644   0.292   0.004   0.085   0.910
B P  3BIWA                 11    0.093  13.225   0.898   0.004   0.420   0.576
E L  3BIWA                 12    0.230  42.095   1.112   0.001   0.900   0.099
B V  3BIWA                 13    0.048   7.316   0.658   0.001   0.959   0.040
E T  3BIWA                 14    0.361  50.126   1.772   0.001   0.959   0.040
B T  3BIWA                 15    0.034   4.771   0.513   0.002   0.816   0.182
E N  3BIWA                 16    0.625  91.471   0.147   0.005   0.262   0.733
E F  3BIWA                 17    0.537 107.736   0.165   0.004   0.085   0.910
B G  3BIWA                 18    0.068   5.320  -1.138   0.004   0.616   0.381
E K  3BIWA                 19    0.335  68.827   1.290   0.001   0.900   0.099
B I  3BIWA                 20    0.031   5.735  -0.398   0.001   0.959   0.040
E R  3BIWA                 21    0.244  55.876   1.708   0.001   0.959   0.040
B G  3BIWA                 22    0.045   3.581  -1.778   0.001   0.900   0.099
E I  3BIWA                 23    0.254  47.027   0.680   0.001   0.959   0.040
B K  3BIWA                 24    0.241  49.677   0.151   0.001   0.959   0.040
B K  3BIWA                 25    0.262  53.893   0.414   0.001   0.900   0.099
E E  3BIWA                 26    0.403  70.404  -0.850   0.021   0.756   0.223
B L  3BIWA                 27    0.157  28.802  -1.017   0.021   0.451   0.528
E N  3BIWA                 28    0.477  69.891  -0.966   0.021   0.279   0.699
E N  3BIWA                 29    0.571  83.521  -2.085   0.019   0.141   0.840
E E  3BIWA                 30    0.451  78.772  -1.660   0.019   0.141   0.840
E I  3BIWA                 31    0.535  98.882  -1.809   0.018   0.088   0.893
E L  3BIWA                 32    0.457  83.677  -1.408   0.018   0.047   0.935
E G  3BIWA                 33    0.508  39.948  -0.539   0.018   0.047   0.935
E P  3BIWA                 34    0.346  49.026   0.779   0.021   0.279   0.699
B V  3BIWA                 35    0.057   8.746   0.528   0.003   0.718   0.279
B I  3BIWA                 36    0.166  30.710   0.544   0.018   0.846   0.136
B Q  3BIWA                 37    0.188  33.613   0.734   0.011   0.918   0.071
B F  3BIWA                 38    0.052  10.436  -0.440   0.018   0.846   0.136
B L  3BIWA                 39    0.212  38.854   0.600   0.022   0.552   0.426
B G  3BIWA                 40    0.261  20.517  -0.988   0.004   0.197   0.799
B V  3BIWA                 41    0.059   9.099   1.246   0.019   0.141   0.840
E P  3BIWA                 42    0.276  39.150   0.258   0.019   0.141   0.840
B Y  3BIWA                 43    0.159  34.085  -0.622   0.019   0.141   0.840
B A  3BIWA                 44    0.076   8.397  -0.984   0.052   0.084   0.864
E A  3BIWA                 45    0.559  61.646   1.197   0.018   0.047   0.935
B P  3BIWA                 46    0.249  35.361   0.504   0.018   0.019   0.964
B P  3BIWA                 47    0.301  42.684  -0.704   0.018   0.019   0.964
E T  3BIWA                 48    0.390  54.107  -0.691   0.058   0.017   0.925
E G  3BIWA                 49    0.443  34.848  -0.454   0.058   0.017   0.925
E E  3BIWA                 50    0.771 134.694   1.139   0.115   0.016   0.868
B H  3BIWA                 51    0.264  48.040   0.204   0.115   0.016   0.868
B R  3BIWA                 52    0.220  50.449   0.511   0.113   0.043   0.844
B F  3BIWA                 53    0.110  21.997  -0.165   0.052   0.084   0.864
E Q  3BIWA                 54    0.477  85.264   1.288   0.052   0.084   0.864
E P  3BIWA                 55    0.446  63.344   0.679   0.018   0.047   0.935
B P  3BIWA                 56    0.151  21.370  -0.347   0.018   0.047   0.935
E E  3BIWA                 57    0.437  76.414   0.130   0.018   0.047   0.935
E P  3BIWA                 58    0.447  63.373   0.421   0.018   0.047   0.935
E P  3BIWA                 59    0.293  41.563   0.157   0.018   0.019   0.964
E S  3BIWA                 60    0.671  78.653  -0.306   0.018   0.019   0.964
E P  3BIWA                 61    0.570  80.840  -0.112   0.018   0.019   0.964
B W  3BIWA                 62    0.166  39.803  -1.223   0.018   0.019   0.964
E S  3BIWA                 63    0.690  80.833   0.015   0.058   0.017   0.925
E D  3BIWA                 64    0.447  64.369  -0.408   0.053   0.043   0.903
B I  3BIWA                 65    0.260  48.026  -0.411   0.066   0.296   0.638
B R  3BIWA                 66    0.224  51.388   1.142   0.022   0.552   0.426
E N  3BIWA                 67    0.317  46.423   0.408   0.021   0.451   0.528
B A  3BIWA                 68    0.021   2.336   0.141   0.064   0.216   0.721
E T  3BIWA                 69    0.364  50.459  -0.044   0.056   0.142   0.802
E Q  3BIWA                 70    0.546  97.533  -0.148   0.056   0.142   0.802
B F  3BIWA                 71    0.294  59.066  -0.119   0.018   0.088   0.893
B A  3BIWA                 72    0.274  30.140  -0.845   0.018   0.019   0.964
E P  3BIWA                 73    0.395  56.121  -1.700   0.018   0.019   0.964
B V  3BIWA                 74    0.285  43.743  -0.600   0.018   0.088   0.893
B C  3BIWA                 75    0.022   3.117   0.030   0.018   0.047   0.935
B P  3BIWA                 76    0.103  14.602  -0.829   0.018   0.088   0.893
B Q  3BIWA                 77    0.049   8.805  -1.416   0.018   0.088   0.893
E N  3BIWA                 78    0.328  47.961  -0.874   0.018   0.088   0.893
E I  3BIWA                 79    0.327  60.421  -1.672   0.018   0.047   0.935
E I  3BIWA                 80    0.395  73.075  -1.962   0.058   0.017   0.925
E D  3BIWA                 81    0.394  56.819  -2.550   0.115   0.016   0.868
E G  3BIWA                 82    0.443  34.888  -2.311   0.115   0.016   0.868
E R  3BIWA                 83    0.429  98.310  -2.035   0.115   0.016   0.868
E L  3BIWA                 84    0.420  76.902  -2.116   0.181   0.016   0.803
E P  3BIWA                 85    0.389  55.142  -2.327   0.181   0.016   0.803
E E  3BIWA                 86    0.342  59.712  -1.924   0.257   0.016   0.727
E V  3BIWA                 87    0.438  67.290  -2.083   0.257   0.016   0.727
E M  3BIWA                 88    0.343  68.734  -1.735   0.339   0.016   0.645
B L  3BIWA                 89    0.234  42.809  -2.062   0.354   0.048   0.598
B P  3BIWA                 90    0.298  42.329  -2.006   0.354   0.048   0.598
E V  3BIWA                 91    0.365  56.131  -1.788   0.386   0.097   0.517
B W  3BIWA                 92    0.292  70.298  -1.677   0.278   0.093   0.628
B F  3BIWA                 93    0.281  56.377  -1.464   0.278   0.093   0.628
E T  3BIWA                 94    0.319  44.273  -1.949   0.268   0.043   0.689
E N  3BIWA                 95    0.404  59.219  -2.109   0.268   0.043   0.689
E N  3BIWA                 96    0.393  57.579  -1.839   0.268   0.043   0.689
B L  3BIWA                 97    0.260  47.551  -1.986   0.268   0.043   0.689
E D  3BIWA                 98    0.373  53.706  -1.959   0.184   0.043   0.773
B V  3BIWA                 99    0.278  42.652  -2.030   0.184   0.043   0.773
B V  3BIWA                100    0.225  34.521  -2.558   0.184   0.043   0.773
E S  3BIWA                101    0.389  45.567  -2.041   0.113   0.043   0.844
E S  3BIWA                102    0.484  56.725  -1.887   0.113   0.087   0.800
E Y  3BIWA                103    0.414  88.493  -1.680   0.052   0.084   0.864
E V  3BIWA                104    0.403  61.972  -1.756   0.056   0.142   0.802
E Q  3BIWA                105    0.410  73.297  -1.165   0.020   0.205   0.775
E D  3BIWA                106    0.451  65.047  -1.372   0.020   0.205   0.775
E Q  3BIWA                107    0.372  66.439  -1.308   0.019   0.141   0.840
B S  3BIWA                108    0.208  24.342  -1.860   0.018   0.088   0.893
E E  3BIWA                109    0.310  54.105  -0.678   0.004   0.085   0.910
B D  3BIWA                110    0.222  32.019  -0.205   0.004   0.138   0.858
B C  3BIWA                111    0.029   4.015   0.890   0.004   0.420   0.576
B L  3BIWA                112    0.027   4.962   0.312   0.002   0.816   0.182
B Y  3BIWA                113    0.089  18.998   0.447   0.001   0.959   0.040
B L  3BIWA                114    0.016   2.930   1.505   0.001   0.959   0.040
B N  3BIWA                115    0.027   4.026   0.411   0.001   0.900   0.099
B I  3BIWA                116    0.017   3.127   1.093   0.001   0.900   0.099
B Y  3BIWA                117    0.062  13.228   0.098   0.002   0.816   0.182
B V  3BIWA                118    0.051   7.808   0.762   0.004   0.514   0.481
B P  3BIWA                119    0.064   9.096  -0.048   0.020   0.205   0.775
E T  3BIWA                120    0.431  59.821   0.206   0.018   0.047   0.935
E E  3BIWA                121    0.466  81.428  -0.542   0.018   0.019   0.964
E D  3BIWA                122    0.403  58.072  -0.786   0.018   0.019   0.964
E D  3BIWA                123    0.575  82.872  -1.871   0.058   0.017   0.925
E I  3BIWA                124    0.578 106.948  -1.846   0.058   0.017   0.925
E R  3BIWA                125    0.582 133.209  -1.086   0.058   0.017   0.925
E D  3BIWA                126    0.458  66.027  -0.795   0.058   0.017   0.925
E S  3BIWA                127    0.600  70.285  -1.203   0.018   0.019   0.964
E G  3BIWA                128    0.725  57.089  -1.762   0.016   0.005   0.979
E G  3BIWA                129    0.458  36.021   0.098   0.018   0.019   0.964
E P  3BIWA                130    0.369  52.290   0.577   0.005   0.015   0.979
B K  3BIWA                131    0.070  14.378   1.299   0.004   0.085   0.910
B P  3BIWA                132    0.031   4.399   0.418   0.004   0.420   0.576
B V  3BIWA                133    0.017   2.628   1.272   0.001   0.900   0.099
B M  3BIWA                134    0.016   3.262   1.694   0.001   0.959   0.040
B V  3BIWA                135    0.013   1.967   1.644   0.001   0.959   0.040
B Y  3BIWA                136    0.022   4.616   1.256   0.001   0.959   0.040
B I  3BIWA                137    0.017   3.127   1.327   0.002   0.816   0.182
B H  3BIWA                138    0.054   9.914   0.648   0.005   0.262   0.733
B G  3BIWA                139    0.041   3.242  -1.984   0.018   0.019   0.964
B G  3BIWA                140    0.065   5.100  -1.157   0.018   0.019   0.964
B S  3BIWA                141    0.066   7.723  -2.132   0.052   0.084   0.864
B Y  3BIWA                142    0.061  13.100  -0.333   0.064   0.216   0.721
B M  3BIWA                143    0.108  21.691  -0.890   0.021   0.279   0.699
B E  3BIWA                144    0.158  27.672  -1.100   0.021   0.279   0.699
B G  3BIWA                145    0.057   4.454  -3.296   0.019   0.141   0.840
B T  3BIWA                146    0.108  14.966  -1.941   0.018   0.047   0.935
B G  3BIWA                147    0.088   6.965  -2.618   0.018   0.019   0.964
B N  3BIWA                148    0.254  37.244  -1.482   0.018   0.019   0.964
B L  3BIWA                149    0.203  37.224  -1.797   0.018   0.047   0.935
B Y  3BIWA                150    0.121  25.943  -0.906   0.018   0.047   0.935
E D  3BIWA                151    0.253  36.457  -0.919   0.058   0.017   0.925
B G  3BIWA                152    0.076   5.989  -1.829   0.339   0.016   0.645
E S  3BIWA                153    0.382  44.817   0.280   0.561   0.047   0.393
B V  3BIWA                154    0.253  38.932   0.405   0.561   0.047   0.393
B L  3BIWA                155    0.032   5.786   0.080   0.561   0.047   0.393
B A  3BIWA                156    0.027   3.019  -0.269   0.561   0.047   0.393
E S  3BIWA                157    0.488  57.158   0.585   0.386   0.097   0.517
E Y  3BIWA                158    0.477 101.935   0.312   0.053   0.043   0.903
E G  3BIWA                159    0.315  24.822   0.154   0.005   0.015   0.979
B N  3BIWA                160    0.201  29.368  -0.440   0.005   0.045   0.951
B V  3BIWA                161    0.026   4.073   0.963   0.001   0.900   0.099
B I  3BIWA                162    0.022   4.014   0.762   0.001   0.959   0.040
B V  3BIWA                163    0.027   4.196   0.973   0.000   0.983   0.017
B I  3BIWA                164    0.014   2.553   1.493   0.000   0.983   0.017
B T  3BIWA                165    0.029   3.967   0.705   0.001   0.959   0.040
B V  3BIWA                166    0.026   4.042   0.723   0.002   0.816   0.182
B N  3BIWA                167    0.050   7.379  -0.276   0.022   0.359   0.619
B Y  3BIWA                168    0.066  14.147   0.198   0.118   0.150   0.732
B R  3BIWA                169    0.052  11.862  -0.762   0.191   0.086   0.723
B L  3BIWA                170    0.035   6.482  -0.296   0.354   0.048   0.598
B G  3BIWA                171    0.034   2.707  -0.739   0.354   0.048   0.598
B V  3BIWA                172    0.044   6.778  -0.855   0.268   0.043   0.689
B L  3BIWA                173    0.044   8.038  -0.978   0.191   0.086   0.723
B G  3BIWA                174    0.027   2.109  -1.592   0.064   0.216   0.721
B F  3BIWA                175    0.072  14.470  -0.488   0.064   0.216   0.721
B L  3BIWA                176    0.064  11.755  -1.016   0.056   0.142   0.802
B S  3BIWA                177    0.230  26.956  -1.111   0.018   0.088   0.893
B T  3BIWA                178    0.104  14.453  -0.996   0.018   0.047   0.935
E G  3BIWA                179    0.540  42.522  -0.939   0.018   0.019   0.964
E D  3BIWA                180    0.612  88.132  -1.433   0.018   0.019   0.964
E Q  3BIWA                181    0.556  99.302  -1.684   0.058   0.017   0.925
E A  3BIWA                182    0.635  69.999  -1.581   0.058   0.017   0.925
B A  3BIWA                183    0.209  22.999  -0.166   0.053   0.043   0.903
E K  3BIWA                184    0.443  91.063  -0.412   0.053   0.043   0.903
B G  3BIWA                185    0.193  15.173  -1.158   0.184   0.043   0.773
B N  3BIWA                186    0.079  11.624  -1.952   0.184   0.043   0.773
B Y  3BIWA                187    0.111  23.721   0.606   0.430   0.016   0.555
B G  3BIWA                188    0.053   4.179  -0.961   0.522   0.016   0.462
B L  3BIWA                189    0.046   8.386   0.039   0.879   0.010   0.111
B L  3BIWA                190    0.136  24.847   0.814   0.923   0.002   0.076
B D  3BIWA                191    0.038   5.533  -0.535   0.970   0.001   0.030
B L  3BIWA                192    0.022   3.992   1.189   0.988   0.000   0.012
B I  3BIWA                193    0.093  17.205   0.487   0.988   0.000   0.012
B Q  3BIWA                194    0.058  10.270   0.671   0.988   0.000   0.012
B A  3BIWA                195    0.017   1.884   0.882   0.988   0.000   0.012
B L  3BIWA                196    0.031   5.768   0.310   0.970   0.001   0.030
E R  3BIWA                197    0.325  74.379   1.831   0.988   0.000   0.012
B W  3BIWA                198    0.058  13.973   0.711   0.970   0.001   0.030
B T  3BIWA                199    0.019   2.608   1.397   0.970   0.001   0.030
E S  3BIWA                200    0.284  33.332   1.016   0.970   0.001   0.030
E E  3BIWA                201    0.488  85.306   1.473   0.970   0.001   0.030
B N  3BIWA                202    0.069  10.146   1.068   0.923   0.002   0.076
B I  3BIWA                203    0.031   5.772   0.715   0.923   0.002   0.076
E G  3BIWA                204    0.520  40.893   0.684   0.858   0.002   0.139
E F  3BIWA                205    0.469  94.068  -0.075   0.694   0.003   0.303
B F  3BIWA                206    0.093  18.725  -0.554   0.321   0.003   0.675
B G  3BIWA                207    0.259  20.352  -2.047   0.053   0.005   0.942
B G  3BIWA                208    0.200  15.716  -2.142   0.016   0.005   0.979
B D  3BIWA                209    0.223  32.149  -0.029   0.018   0.019   0.964
B P  3BIWA                210    0.268  37.972  -0.333   0.018   0.019   0.964
E L  3BIWA                211    0.581 106.454   0.070   0.018   0.019   0.964
E R  3BIWA                212    0.230  52.647   1.418   0.020   0.205   0.775
B I  3BIWA                213    0.020   3.718   1.261   0.021   0.756   0.223
B T  3BIWA                214    0.040   5.506   0.469   0.011   0.918   0.071
B V  3BIWA                215    0.016   2.521   1.135   0.011   0.918   0.071
B F  3BIWA                216    0.033   6.623   0.246   0.011   0.918   0.071
B G  3BIWA                217    0.020   1.574  -0.061   0.003   0.718   0.279
B S  3BIWA                218    0.053   6.270  -0.203   0.004   0.514   0.481
B G  3BIWA                219    0.037   2.936  -1.459   0.052   0.084   0.864
B A  3BIWA                220    0.055   6.116  -1.061   0.430   0.016   0.555
B G  3BIWA                221    0.026   2.070  -0.948   0.694   0.003   0.303
B G  3BIWA                222    0.020   1.543  -0.657   0.858   0.002   0.139
B S  3BIWA                223    0.051   6.024  -1.563   0.879   0.010   0.111
B C  3BIWA                224    0.021   2.948   0.427   0.938   0.007   0.055
B V  3BIWA                225    0.017   2.613   1.011   0.938   0.007   0.055
B N  3BIWA                226    0.042   6.149  -0.552   0.938   0.007   0.055
B L  3BIWA                227    0.032   5.914  -0.010   0.938   0.007   0.055
B L  3BIWA                228    0.025   4.596   0.826   0.879   0.010   0.111
B T  3BIWA                229    0.032   4.438  -0.480   0.717   0.014   0.269
B L  3BIWA                230    0.044   8.038  -0.371   0.502   0.002   0.495
B S  3BIWA                231    0.039   4.536  -0.293   0.176   0.004   0.820
B H  3BIWA                232    0.150  27.285   0.034   0.246   0.004   0.750
E Y  3BIWA                233    0.437  93.387  -1.129   0.109   0.005   0.886
B S  3BIWA                234    0.179  20.955  -1.461   0.053   0.005   0.942
B E  3BIWA                235    0.240  41.998  -1.588   0.176   0.004   0.820
E K  3BIWA                236    0.402  82.712  -0.065   0.406   0.004   0.590
E G  3BIWA                237    0.257  20.257  -1.106   0.600   0.003   0.397
B L  3BIWA                238    0.065  11.865   0.729   0.802   0.014   0.185
B F  3BIWA                239    0.018   3.673   0.905   0.831   0.044   0.125
B Q  3BIWA                240    0.126  22.557   0.526   0.779   0.100   0.120
B R  3BIWA                241    0.102  23.427   0.385   0.807   0.137   0.056
B A  3BIWA                242    0.017   1.862   1.347   0.807   0.137   0.056
B I  3BIWA                243    0.020   3.756   1.285   0.662   0.225   0.113
B A  3BIWA                244    0.025   2.700   0.066   0.478   0.309   0.214
B Q  3BIWA                245    0.052   9.287  -0.272   0.321   0.252   0.427
B S  3BIWA                246    0.024   2.860  -0.791   0.113   0.087   0.800
B G  3BIWA                247    0.051   4.014  -1.735   0.053   0.043   0.903
B T  3BIWA                248    0.090  12.497  -1.545   0.053   0.043   0.903
B A  3BIWA                249    0.060   6.667  -1.956   0.053   0.043   0.903
B L  3BIWA                250    0.190  34.789  -1.904   0.058   0.017   0.925
B S  3BIWA                251    0.113  13.220  -1.861   0.058   0.017   0.925
B S  3BIWA                252    0.251  29.441  -1.772   0.058   0.017   0.925
B W  3BIWA                253    0.222  53.295  -1.657   0.113   0.043   0.844
B A  3BIWA                254    0.299  32.906  -1.638   0.058   0.017   0.925
B V  3BIWA                255    0.213  32.738  -0.721   0.058   0.017   0.925
E S  3BIWA                256    0.319  37.352  -0.283   0.257   0.016   0.727
E F  3BIWA                257    0.637 127.946  -0.220   0.600   0.003   0.397
E Q  3BIWA                258    0.496  88.621   0.472   0.694   0.003   0.303
B P  3BIWA                259    0.054   7.620  -1.178   0.923   0.002   0.076
E A  3BIWA                260    0.279  30.713   0.786   0.970   0.001   0.030
E K  3BIWA                261    0.546 112.394   1.227   0.970   0.001   0.030
B Y  3BIWA                262    0.151  32.226   1.659   0.970   0.001   0.030
B A  3BIWA                263    0.015   1.675   1.316   0.988   0.000   0.012
E R  3BIWA                264    0.334  76.578   1.835   0.988   0.000   0.012
E I  3BIWA                265    0.372  68.820   2.100   0.988   0.000   0.012
B L  3BIWA                266    0.030   5.511   0.672   0.988   0.000   0.012
B A  3BIWA                267    0.022   2.380   0.450   0.970   0.001   0.030
E T  3BIWA                268    0.632  87.686   1.727   0.970   0.001   0.030
E K  3BIWA                269    0.444  91.290   1.083   0.923   0.002   0.076
B V  3BIWA                270    0.102  15.724   0.847   0.502   0.002   0.495
E G  3BIWA                271    0.711  55.948  -0.534   0.016   0.005   0.979
B C  3BIWA                272    0.104  14.588   0.268   0.003   0.003   0.994
E N  3BIWA                273    0.668  97.781   0.116   0.003   0.003   0.994
E V  3BIWA                274    0.472  72.470  -1.124   0.003   0.003   0.994
E S  3BIWA                275    0.601  70.425  -1.478   0.003   0.003   0.994
E D  3BIWA                276    0.537  77.411  -0.165   0.003   0.003   0.994
E T  3BIWA                277    0.244  33.857  -0.281   0.782   0.003   0.216
E V  3BIWA                278    0.562  86.441   0.639   0.923   0.002   0.076
E E  3BIWA                279    0.470  82.057   1.730   0.923   0.002   0.076
B L  3BIWA                280    0.069  12.616   0.568   0.970   0.001   0.030
B V  3BIWA                281    0.087  13.449   0.952   0.970   0.001   0.030
E E  3BIWA                282    0.536  93.692   1.955   0.970   0.001   0.030
B C  3BIWA                283    0.144  20.190   1.085   0.923   0.002   0.076
B L  3BIWA                284    0.031   5.676   0.312   0.858   0.002   0.139
E Q  3BIWA                285    0.318  56.723   0.768   0.694   0.003   0.303
E K  3BIWA                286    0.813 167.296   1.510   0.246   0.004   0.750
B K  3BIWA                287    0.137  28.181   1.853   0.016   0.005   0.979
E P  3BIWA                288    0.411  58.264   1.441   0.005   0.015   0.979
B Y  3BIWA                289    0.085  18.122   0.017   0.923   0.002   0.076
E K  3BIWA                290    0.607 124.901   1.503   0.970   0.001   0.030
E E  3BIWA                291    0.334  58.332   2.361   0.970   0.001   0.030
B L  3BIWA                292    0.022   4.065   1.067   0.970   0.001   0.030
B V  3BIWA                293    0.169  25.975   0.964   0.970   0.001   0.030
E D  3BIWA                294    0.572  82.411   1.725   0.923   0.002   0.076
B Q  3BIWA                295    0.099  17.664   0.331   0.858   0.002   0.139
B D  3BIWA                296    0.143  20.635  -0.387   0.782   0.003   0.216
E V  3BIWA                297    0.441  67.766  -0.871   0.694   0.003   0.303
E Q  3BIWA                298    0.431  76.959  -0.397   0.600   0.003   0.397
B P  3BIWA                299    0.142  20.136  -1.048   0.406   0.004   0.590
E A  3BIWA                300    0.400  44.135  -2.015   0.181   0.016   0.803
E R  3BIWA                301    0.400  91.508  -2.438   0.058   0.017   0.925
B Y  3BIWA                302    0.214  45.732  -2.090   0.018   0.047   0.935
B H  3BIWA                303    0.228  41.419  -2.266   0.018   0.088   0.893
B I  3BIWA                304    0.067  12.432  -1.481   0.004   0.197   0.799
B A  3BIWA                305    0.069   7.637  -2.028   0.005   0.336   0.660
B F  3BIWA                306    0.039   7.888  -0.709   0.004   0.514   0.481
B G  3BIWA                307    0.059   4.604  -0.129   0.004   0.514   0.481
B P  3BIWA                308    0.025   3.604   0.095   0.005   0.336   0.660
B V  3BIWA                309    0.043   6.671  -0.472   0.021   0.451   0.528
B I  3BIWA                310    0.107  19.758  -0.129   0.004   0.420   0.576
B D  3BIWA                311    0.195  28.071  -0.170   0.018   0.088   0.893
E G  3BIWA                312    0.350  27.529  -0.583   0.018   0.047   0.935
E D  3BIWA                313    0.422  60.781   0.288   0.052   0.084   0.864
B V  3BIWA                314    0.113  17.322   0.263   0.056   0.142   0.802
B I  3BIWA                315    0.035   6.494   0.829   0.020   0.205   0.775
B P  3BIWA                316    0.254  36.099   0.581   0.019   0.141   0.840
E D  3BIWA                317    0.548  78.938   0.806   0.115   0.016   0.868
E D  3BIWA                318    0.360  51.862   1.193   0.115   0.016   0.868
B P  3BIWA                319    0.050   7.081  -0.066   0.923   0.002   0.076
E Q  3BIWA                320    0.265  47.293   0.623   0.970   0.001   0.030
E I  3BIWA                321    0.435  80.401   1.477   0.970   0.001   0.030
B L  3BIWA                322    0.047   8.587  -0.090   0.970   0.001   0.030
B M  3BIWA                323    0.073  14.527  -0.970   0.923   0.002   0.076
E E  3BIWA                324    0.545  95.281   0.393   0.858   0.002   0.139
E Q  3BIWA                325    0.530  94.604   0.233   0.406   0.004   0.590
E G  3BIWA                326    0.463  36.414  -1.344   0.109   0.005   0.886
E E  3BIWA                327    0.520  90.757  -0.579   0.016   0.005   0.979
B F  3BIWA                328    0.121  24.285  -1.301   0.018   0.019   0.964
E L  3BIWA                329    0.364  66.575  -0.089   0.018   0.019   0.964
E N  3BIWA                330    0.511  74.869  -0.726   0.018   0.019   0.964
B Y  3BIWA                331    0.118  25.195   0.803   0.018   0.019   0.964
B D  3BIWA                332    0.083  11.903  -2.052   0.018   0.088   0.893
B I  3BIWA                333    0.044   8.122   0.035   0.021   0.756   0.223
B M  3BIWA                334    0.015   3.022   0.667   0.011   0.918   0.071
B L  3BIWA                335    0.025   4.578   0.440   0.011   0.918   0.071
B G  3BIWA                336    0.016   1.251  -0.045   0.018   0.846   0.136
B V  3BIWA                337    0.043   6.640   0.397   0.003   0.718   0.279
B N  3BIWA                338    0.019   2.752   0.934   0.022   0.359   0.619
E Q  3BIWA                339    0.172  30.737   0.376   0.113   0.087   0.800
B G  3BIWA                340    0.163  12.820  -1.676   0.181   0.016   0.803
B E  3BIWA                341    0.043   7.530  -1.787   0.257   0.016   0.727
B G  3BIWA                342    0.048   3.778  -2.358   0.268   0.043   0.689
B L  3BIWA                343    0.089  16.278  -0.808   0.216   0.235   0.548
B K  3BIWA                344    0.074  15.140  -0.776   0.231   0.330   0.439
B F  3BIWA                345    0.076  15.293  -0.728   0.321   0.252   0.427
B V  3BIWA                346    0.078  12.019  -1.222   0.307   0.165   0.527
B E  3BIWA                347    0.185  32.337  -1.797   0.307   0.165   0.527
B N  3BIWA                348    0.253  37.068  -1.431   0.278   0.093   0.628
B I  3BIWA                349    0.177  32.689  -1.984   0.278   0.093   0.628
B V  3BIWA                350    0.246  37.856  -1.183   0.268   0.043   0.689
E D  3BIWA                351    0.479  69.082  -1.633   0.257   0.016   0.727
E S  3BIWA                352    0.306  35.828  -1.149   0.321   0.003   0.675
E D  3BIWA                353    0.373  53.749  -1.700   0.502   0.002   0.495
E D  3BIWA                354    0.519  74.730  -1.653   0.600   0.003   0.397
E G  3BIWA                355    0.346  27.207  -1.413   0.622   0.015   0.363
B V  3BIWA                356    0.160  24.577  -1.959   0.622   0.015   0.363
E S  3BIWA                357    0.349  40.950  -0.921   0.622   0.015   0.363
E A  3BIWA                358    0.431  47.463  -0.890   0.717   0.014   0.269
E S  3BIWA                359    0.317  37.141  -1.057   0.717   0.014   0.269
B D  3BIWA                360    0.247  35.650  -1.126   0.717   0.014   0.269
B F  3BIWA                361    0.209  42.027  -0.378   0.802   0.014   0.185
E D  3BIWA                362    0.392  56.487  -1.310   0.782   0.003   0.216
E F  3BIWA                363    0.339  68.037  -0.345   0.782   0.003   0.216
B A  3BIWA                364    0.123  13.555  -0.848   0.782   0.003   0.216
B V  3BIWA                365    0.071  10.990  -0.199   0.858   0.002   0.139
E S  3BIWA                366    0.372  43.622  -0.165   0.858   0.002   0.139
E N  3BIWA                367    0.354  51.767   0.236   0.858   0.002   0.139
B F  3BIWA                368    0.070  14.009  -0.492   0.858   0.002   0.139
B V  3BIWA                369    0.077  11.820  -0.940   0.782   0.003   0.216
E D  3BIWA                370    0.406  58.562  -0.474   0.782   0.003   0.216
B N  3BIWA                371    0.267  39.133  -0.979   0.782   0.003   0.216
B L  3BIWA                372    0.138  25.304  -1.158   0.694   0.003   0.303
B Y  3BIWA                373    0.285  60.883  -0.873   0.502   0.002   0.495
E G  3BIWA                374    0.451  35.509  -1.387   0.246   0.004   0.750
B Y  3BIWA                375    0.193  41.308  -0.356   0.115   0.016   0.868
E P  3BIWA                376    0.481  68.226  -1.541   0.181   0.016   0.803
E E  3BIWA                377    0.619 108.157  -1.426   0.246   0.004   0.750
E G  3BIWA                378    0.317  24.948  -1.378   0.321   0.003   0.675
E K  3BIWA                379    0.372  76.418  -0.415   0.694   0.003   0.303
E D  3BIWA                380    0.576  83.059  -0.363   0.858   0.002   0.139
E V  3BIWA                381    0.239  36.811   0.078   0.923   0.002   0.076
B L  3BIWA                382    0.048   8.734  -0.570   0.923   0.002   0.076
E R  3BIWA                383    0.252  57.731   0.258   0.970   0.001   0.030
E E  3BIWA                384    0.459  80.275   0.623   0.970   0.001   0.030
B T  3BIWA                385    0.075  10.458  -0.481   0.970   0.001   0.030
B I  3BIWA                386    0.042   7.789  -0.016   0.923   0.002   0.076
E K  3BIWA                387    0.347  71.460   0.823   0.923   0.002   0.076
E F  3BIWA                388    0.336  67.415  -0.362   0.858   0.002   0.139
B M  3BIWA                389    0.112  22.351   0.292   0.782   0.003   0.216
B Y  3BIWA                390    0.128  27.311  -0.770   0.600   0.003   0.397
E T  3BIWA                391    0.409  56.701  -1.468   0.502   0.002   0.495
E D  3BIWA                392    0.435  62.626  -1.723   0.339   0.016   0.645
B W  3BIWA                393    0.213  51.347  -1.811   0.176   0.004   0.820
E A  3BIWA                394    0.504  55.497  -1.871   0.109   0.005   0.886
E D  3BIWA                395    0.577  83.160  -2.503   0.053   0.005   0.942
E R  3BIWA                396    0.514 117.798  -1.563   0.053   0.005   0.942
E H  3BIWA                397    0.542  98.535  -1.902   0.016   0.005   0.979
E N  3BIWA                398    0.297  43.437  -1.062   0.053   0.005   0.942
E P  3BIWA                399    0.293  41.577  -1.594   0.502   0.002   0.495
E E  3BIWA                400    0.564  98.601  -0.896   0.858   0.002   0.139
E T  3BIWA                401    0.481  66.770   0.413   0.858   0.002   0.139
B R  3BIWA                402    0.078  17.908  -0.098   0.923   0.002   0.076
B R  3BIWA                403    0.085  19.419   0.525   0.970   0.001   0.030
E K  3BIWA                404    0.427  87.854   1.247   0.970   0.001   0.030
B T  3BIWA                405    0.158  21.928   0.337   0.988   0.000   0.012
B L  3BIWA                406    0.024   4.449   0.698   0.988   0.000   0.012
B L  3BIWA                407    0.047   8.642  -0.087   0.988   0.000   0.012
E A  3BIWA                408    0.247  27.252   0.991   0.970   0.001   0.030
B L  3BIWA                409    0.034   6.244   0.089   0.970   0.001   0.030
B F  3BIWA                410    0.053  10.577   0.269   0.938   0.007   0.055
B T  3BIWA                411    0.055   7.573  -1.231   0.782   0.003   0.216
B D  3BIWA                412    0.083  11.975  -1.171   0.600   0.003   0.397
B H  3BIWA                413    0.068  12.387  -0.049   0.600   0.003   0.397
B Q  3BIWA                414    0.114  20.414  -0.214   0.622   0.015   0.363
B W  3BIWA                415    0.177  42.641  -0.393   0.622   0.015   0.363
B V  3BIWA                416    0.097  14.863  -0.699   0.622   0.015   0.363
B A  3BIWA                417    0.068   7.505   0.393   0.430   0.016   0.555
B P  3BIWA                418    0.105  14.956  -0.681   0.622   0.015   0.363
B A  3BIWA                419    0.056   6.138  -0.833   0.858   0.002   0.139
B V  3BIWA                420    0.152  23.409   0.210   0.970   0.001   0.030
B A  3BIWA                421    0.177  19.472   0.559   0.970   0.001   0.030
B T  3BIWA                422    0.047   6.505   0.053   0.970   0.001   0.030
B A  3BIWA                423    0.048   5.334  -0.259   0.970   0.001   0.030
E D  3BIWA                424    0.368  53.029   1.103   0.970   0.001   0.030
B L  3BIWA                425    0.149  27.319   0.454   0.970   0.001   0.030
B H  3BIWA                426    0.105  19.045   0.353   0.923   0.002   0.076
E S  3BIWA                427    0.274  32.136  -0.191   0.923   0.002   0.076
E N  3BIWA                428    0.649  95.072   0.029   0.782   0.003   0.216
E F  3BIWA                429    0.357  71.590  -0.695   0.321   0.003   0.675
E G  3BIWA                430    0.668  52.556  -1.555   0.018   0.019   0.964
B S  3BIWA                431    0.237  27.730  -0.706   0.005   0.015   0.979
E P  3BIWA                432    0.328  46.572  -0.168   0.004   0.138   0.858
B T  3BIWA                433    0.043   5.964   0.166   0.003   0.718   0.279
B Y  3BIWA                434    0.066  14.104   0.047   0.001   0.900   0.099
B F  3BIWA                435    0.041   8.309  -0.068   0.001   0.959   0.040
B Y  3BIWA                436    0.021   4.573   1.170   0.001   0.959   0.040
B A  3BIWA                437    0.073   8.045   0.460   0.001   0.959   0.040
B F  3BIWA                438    0.024   4.857   0.933   0.001   0.900   0.099
B Y  3BIWA                439    0.106  22.738  -1.033   0.021   0.756   0.223
B H  3BIWA                440    0.094  17.117   0.221   0.004   0.616   0.381
E H  3BIWA                441    0.330  60.082   0.393   0.022   0.359   0.619
B C  3BIWA                442    0.139  19.516  -1.547   0.018   0.088   0.893
E Q  3BIWA                443    0.603 107.785  -2.015   0.018   0.019   0.964
E T  3BIWA                444    0.397  55.050  -2.147   0.018   0.019   0.964
E D  3BIWA                445    0.379  54.556  -2.649   0.018   0.019   0.964
E Q  3BIWA                446    0.527  94.033  -2.536   0.018   0.019   0.964
B V  3BIWA                447    0.271  41.653  -1.368   0.018   0.019   0.964
E P  3BIWA                448    0.421  59.726  -1.560   0.018   0.019   0.964
E A  3BIWA                449    0.445  49.017  -1.484   0.018   0.047   0.935
B W  3BIWA                450    0.200  48.124  -1.701   0.018   0.088   0.893
B A  3BIWA                451    0.135  14.921  -0.978   0.052   0.084   0.864
B D  3BIWA                452    0.080  11.514  -1.372   0.056   0.142   0.802
B A  3BIWA                453    0.018   1.940   0.764   0.056   0.142   0.802
B A  3BIWA                454    0.034   3.791  -0.125   0.135   0.317   0.548
B H  3BIWA                455    0.027   4.839   0.716   0.231   0.330   0.439
B G  3BIWA                456    0.024   1.873  -0.230   0.428   0.171   0.402
B D  3BIWA                457    0.064   9.165  -0.397   0.502   0.102   0.396
B E  3BIWA                458    0.027   4.699   0.267   0.605   0.105   0.290
B V  3BIWA                459    0.016   2.490   1.699   0.649   0.163   0.188
B P  3BIWA                460    0.055   7.776   0.523   0.649   0.163   0.188
B Y  3BIWA                461    0.035   7.522   0.160   0.649   0.163   0.188
B V  3BIWA                462    0.023   3.458   0.467   0.538   0.173   0.289
B L  3BIWA                463    0.024   4.468   0.463   0.428   0.171   0.402
B G  3BIWA                464    0.161  12.647  -1.194   0.191   0.086   0.723
B I  3BIWA                465    0.072  13.357  -0.629   0.184   0.043   0.773
B P  3BIWA                466    0.200  28.337  -0.920   0.113   0.043   0.844
B M  3BIWA                467    0.207  41.441  -1.762   0.181   0.016   0.803
B I  3BIWA                468    0.219  40.478  -1.545   0.181   0.016   0.803
E G  3BIWA                469    0.376  29.623  -1.862   0.181   0.016   0.803
E P  3BIWA                470    0.399  56.547  -2.415   0.181   0.016   0.803
E T  3BIWA                471    0.361  50.140  -1.877   0.181   0.016   0.803
E E  3BIWA                472    0.393  68.657  -2.104   0.181   0.016   0.803
E L  3BIWA                473    0.340  62.291  -1.735   0.181   0.016   0.803
E F  3BIWA                474    0.304  60.912  -1.825   0.115   0.016   0.868
E P  3BIWA                475    0.465  65.927  -1.684   0.058   0.017   0.925
E C  3BIWA                476    0.365  51.218  -1.354   0.018   0.019   0.964
E N  3BIWA                477    0.650  95.204  -1.371   0.005   0.015   0.979
B F  3BIWA                478    0.281  56.357  -0.942   0.005   0.015   0.979
E S  3BIWA                479    0.383  44.946  -0.041   0.005   0.015   0.979
E K  3BIWA                480    0.676 138.971   0.634   0.858   0.002   0.139
E N  3BIWA                481    0.477  69.891   1.159   0.923   0.002   0.076
B D  3BIWA                482    0.048   6.974  -1.538   0.923   0.002   0.076
E V  3BIWA                483    0.289  44.450   1.467   0.970   0.001   0.030
E M  3BIWA                484    0.467  93.427   1.881   0.970   0.001   0.030
B L  3BIWA                485    0.041   7.452   0.535   0.970   0.001   0.030
B S  3BIWA                486    0.027   3.176   0.476   0.970   0.001   0.030
E A  3BIWA                487    0.441  48.587   1.696   0.988   0.000   0.012
B V  3BIWA                488    0.223  34.214   1.534   0.988   0.000   0.012
B V  3BIWA                489    0.030   4.565   0.464   0.988   0.000   0.012
B M  3BIWA                490    0.084  16.828   0.834   0.988   0.000   0.012
E T  3BIWA                491    0.397  55.119   0.895   0.988   0.000   0.012
B Y  3BIWA                492    0.098  20.921   0.961   0.988   0.000   0.012
B W  3BIWA                493    0.038   9.211   0.345   0.988   0.000   0.012
B T  3BIWA                494    0.069   9.612   0.039   0.988   0.000   0.012
B N  3BIWA                495    0.171  25.078   0.022   0.970   0.001   0.030
B F  3BIWA                496    0.044   8.771   0.426   0.938   0.007   0.055
B A  3BIWA                497    0.027   3.008   0.265   0.938   0.007   0.055
E K  3BIWA                498    0.322  66.318   1.050   0.717   0.014   0.269
E T  3BIWA                499    0.300  41.568  -0.076   0.339   0.016   0.645
B G  3BIWA                500    0.140  11.018  -1.203   0.018   0.019   0.964
B D  3BIWA                501    0.175  25.145  -0.504   0.005   0.015   0.979
B P  3BIWA                502    0.069   9.749  -2.306   0.016   0.005   0.979
E N  3BIWA                503    0.321  46.950  -0.640   0.003   0.003   0.994
E Q  3BIWA                504    0.420  75.030  -1.248   0.053   0.005   0.942
E P  3BIWA                505    0.526  74.611  -1.897   0.109   0.005   0.886
E V  3BIWA                506    0.368  56.546  -1.173   0.109   0.005   0.886
E P  3BIWA                507    0.433  61.443  -1.874   0.115   0.016   0.868
E Q  3BIWA                508    0.548  97.944  -1.481   0.115   0.016   0.868
E D  3BIWA                509    0.438  63.159  -1.680   0.115   0.016   0.868
E T  3BIWA                510    0.383  53.191  -1.454   0.115   0.016   0.868
E K  3BIWA                511    0.463  95.198  -1.477   0.113   0.043   0.844
E F  3BIWA                512    0.391  78.494  -1.242   0.113   0.087   0.800
E I  3BIWA                513    0.396  73.186  -1.405   0.191   0.086   0.723
E H  3BIWA                514    0.460  83.638  -1.446   0.191   0.086   0.723
E T  3BIWA                515    0.436  60.473  -1.314   0.191   0.086   0.723
E K  3BIWA                516    0.446  91.742  -1.039   0.184   0.043   0.773
E P  3BIWA                517    0.477  67.757  -1.184   0.115   0.016   0.868
E N  3BIWA                518    0.485  71.033  -1.464   0.115   0.016   0.868
E R  3BIWA                519    0.426  97.577  -1.225   0.113   0.087   0.800
E F  3BIWA                520    0.412  82.708  -1.630   0.056   0.142   0.802
E E  3BIWA                521    0.478  83.454  -1.486   0.056   0.142   0.802
E E  3BIWA                522    0.472  82.546  -1.044   0.019   0.141   0.840
B V  3BIWA                523    0.267  41.038  -0.372   0.019   0.141   0.840
E A  3BIWA                524    0.613  67.564   0.612   0.019   0.141   0.840
B W  3BIWA                525    0.065  15.729  -0.093   0.018   0.088   0.893
E T  3BIWA                526    0.397  55.119   0.586   0.018   0.088   0.893
E R  3BIWA                527    0.535 122.492   0.397   0.019   0.141   0.840
B Y  3BIWA                528    0.163  34.748  -0.123   0.019   0.141   0.840
E S  3BIWA                529    0.384  45.040   0.129   0.004   0.085   0.910
E Q  3BIWA                530    0.590 105.303  -0.682   0.018   0.047   0.935
E K  3BIWA                531    0.770 158.410  -0.731   0.018   0.019   0.964
E D  3BIWA                532    0.635  91.489   0.069   0.018   0.047   0.935
E Q  3BIWA                533    0.291  51.955   0.731   0.019   0.141   0.840
E L  3BIWA                534    0.356  65.147   1.159   0.004   0.514   0.481
B Y  3BIWA                535    0.128  27.354   0.768   0.002   0.816   0.182
B L  3BIWA                536    0.049   8.935   0.920   0.001   0.900   0.099
E H  3BIWA                537    0.303  55.152   0.685   0.001   0.900   0.099
B I  3BIWA                538    0.042   7.789   0.667   0.004   0.616   0.381
E G  3BIWA                539    0.415  32.676  -0.377   0.004   0.085   0.910
E L  3BIWA                540    0.511  93.473  -0.824   0.018   0.019   0.964
E K  3BIWA                541    0.693 142.488   0.476   0.018   0.088   0.893
B P  3BIWA                542    0.202  28.664  -0.322   0.020   0.205   0.775
E R  3BIWA                543    0.429  98.127   0.083   0.021   0.451   0.528
E V  3BIWA                544    0.277  42.560  -0.623   0.021   0.451   0.528
E K  3BIWA                545    0.335  68.971   0.190   0.022   0.359   0.619
E E  3BIWA                546    0.587 102.462  -1.147   0.020   0.205   0.775
E H  3BIWA                547    0.429  78.126  -0.775   0.053   0.043   0.903
B Y  3BIWA                548    0.209  44.706  -0.336   0.058   0.017   0.925
E R  3BIWA                549    0.338  77.310   0.614   0.430   0.016   0.555
E A  3BIWA                550    0.473  52.147   0.833   0.858   0.002   0.139
E N  3BIWA                551    0.514  75.279   0.608   0.858   0.002   0.139
B K  3BIWA                552    0.095  19.480   0.935   0.923   0.002   0.076
B V  3BIWA                553    0.068  10.482   1.289   0.970   0.001   0.030
E N  3BIWA                554    0.486  71.194   1.799   0.970   0.001   0.030
B L  3BIWA                555    0.112  20.526   0.932   0.970   0.001   0.030
B W  3BIWA                556    0.020   4.810   1.334   0.988   0.000   0.012
E L  3BIWA                557    0.339  62.162   1.473   0.970   0.001   0.030
E E  3BIWA                558    0.540  94.251   1.611   0.970   0.001   0.030
B L  3BIWA                559    0.074  13.604   0.842   0.970   0.001   0.030
B V  3BIWA                560    0.054   8.300   0.886   0.923   0.002   0.076
E P  3BIWA                561    0.546  77.520   1.506   0.923   0.002   0.076
E H  3BIWA                562    0.410  74.488   1.547   0.858   0.002   0.139
B L  3BIWA                563    0.061  11.132   0.191   0.694   0.003   0.303
E H  3BIWA                564    0.404  73.506   0.566   0.502   0.002   0.495
E N  3BIWA                565    0.682  99.859   1.050   0.321   0.003   0.675
B L  3BIWA                566    0.238  43.559  -0.543   0.321   0.003   0.675
E N  3BIWA                567    0.541  79.232  -1.129   0.321   0.003   0.675
E D  3BIWA                568    0.470  67.684  -0.018   0.406   0.004   0.590
E H  3BIWA                569    0.394  71.669  -0.333   0.406   0.004   0.590
E H  3BIWA                570    0.505  91.823  -0.086   0.406   0.004   0.590
E H  3BIWA                571    0.482  87.621  -0.245   0.522   0.016   0.462
E H  3BIWA                572    0.472  85.857  -0.493   0.430   0.016   0.555
E H  3BIWA                573    0.436  79.236  -0.138   0.268   0.043   0.689
E H  3BIWA                574    0.760 138.244  -0.795   0.003   0.003   0.994

Explain the output.  Go back.
```

---
